# Multimodal MRI of the hippocampus in Parkinson’s disease with visual hallucinations

**DOI:** 10.1007/s00429-014-0907-5

**Published:** 2014-10-07

**Authors:** Nailin Yao, Charlton Cheung, Shirley Pang, Richard Shek-kwan Chang, Kui Kai Lau, John Suckling, Kevin Yu, Henry Ka-Fung Mak, Siew Eng Chua, Shu-Leong Ho, Grainne M. McAlonan

**Affiliations:** 1 Division of Neurology, Department of Medicine, Queen Mary Hospital, The University of Hong Kong, Pokfulam, Hong Kong; 2 Department of Psychiatry, Queen Mary Hospital, The University of Hong Kong, Pokfulam, Hong Kong; 3 Department of Diagnostic Radiology, The University of Hong Kong, Pokfulam, Hong Kong; 4 State Key Laboratory for Cognitive Sciences, The University of Hong Kong, Pokfulam, Hong Kong; 5 Department of Psychiatry and Behavioural and Clinical Neuroscience Institute, United Kingdom and Cambridge and Peterborough Foundation NHS Trust, University of Cambridge, Cambridge, UK; 6 Department of Forensic and Neurodevelopmental Science, Institute of Psychiatry, King’s College London, London, SE5 8AZ UK

**Keywords:** Functional MRI, DTI, Diffusivity, Multimodal, Visual spatial memory

## Abstract

Visual hallucinations carry poor prognosis in Parkinson’s disease. Here we tested the hypothesis that the hippocampus and visuospatial memory impairment play a central role in the pathology of PD with visual hallucinations. Multimodal magnetic resonance imaging of the brain was carried out in 12 people with PD and visual hallucinations; 15 PD individuals without hallucinations; and 14 healthy controls. Age, gender, cognitive ability, and education level were matched across the three groups. PD patients were taking dopaminergic medication. Hippocampal volume, shape, mean diffusivity (MD), and functional connectivity within the whole brain were examined. Visuospatial memory was compared between groups, and correlations with hippocampal MD, functional connectivity, and the severity of hallucinations were explored. There were no macrostructural differences across groups, but individuals with hallucinations had higher diffusivity in posterior hippocampus than the other two groups. Visuospatial memory was poorer in both PD groups compared to controls, and was correlated with hallucinations. Finally, hippocampal functional connectivity in the visual cortices was lower in those with hallucinations than other groups, and this correlated with visuospatial memory impairment. In contrast, functional connectivity between the hippocampus and default mode network regions and frontal regions was greater in the PD hallucinators compared to other groups. We suggest that hippocampal pathology, which disrupts visuospatial memory, makes a key contribution to visual hallucinations in PD. These findings may pave the way for future studies of imaging biomarkers to measure treatment response in those with PD who are most at risk of poor outcomes.

**Electronic supplementary material:**

The online version of this article (doi:10.1007/s00429-014-0907-5) contains supplementary material, which is available to authorized users.

## Introduction

Visual hallucinations are one of the most common and distressing non-motor problems in Parkinson’s disease (PD) (Rabey 2009; Bernal-Pacheco et al. 2012), and predict dementia and mortality (Fenelon et al. 2000; Aarsland et al. 2007). However their underlying biology remains poorly understood. Hippocampal pathology is associated with visual hallucinations in other psychiatric disorders (Isaacson 2002; Oertel et al. 2007; Vignal et al. 2007). The hippocampus is in a unique position for the conjunction of spatial and non-spatial contextual information (Goodale and Milner 1992) and plays an important role in encoding and retrieval of event memories (Behrendt 2010). In experimental animal models, blocking neuronal activity in one hippocampus in rats modulates activity in the contralateral hippocampus and leads to cognitive disorganization (Olypher et al. 2006). This has led to the suggestion that inappropriate integration of visual information by the hippocampi in psychosis could induce hallucinations instead of reflecting reality (Olypher et al. 2006).

There is a preliminary evidence linking macroscopic changes in the hippocampus to visual hallucinations in Parkinson’s disease. For example, greater numbers of Lewy bodies in medial temporal lobe are found in PD patients with visual hallucinations (Kalaitzakis et al. 2009; Gallagher et al. 2011); and hippocampal atrophy has been reported to be correlated with visual hallucinations (Ibarretxe-Bilbao et al. 2008, 2009). However, no-one has directly examined the pathophysiology of the hippocampus in PD patients with visual hallucinations. The present study adopted a multimodal MRI approach to test the hypothesis that structural and functional alterations of the hippocampal formation contribute to visual hallucinations in PD. We compared hippocampal volume, shape, gray matter microstructure, and functional connectivity in individuals with PD with and without visual hallucinations, and healthy controls (HC). We also compared visuospatial memory function in each group because this ability depends upon the hippocampus (Dupret et al. 2010) and impaired visuospatial function is, in turn, linked to visual hallucinations (Barnes and Boubert 2011).

Diffusion tensor imaging (DTI) is a non-invasive measure of microstructural integrity of tissue (Taylor and Bushell 1985; Merboldt et al. 1991). Although it has been primarily used to detect regional white matter differences, microstructural anomalies of gray matter can also be examined using DTI. This approach has been successfully applied to investigate the organization of subcortical regions (Cherubini et al. 2010) and hippocampus (Muller et al. 2005, 2007; Carlesimo et al. 2012). We predicted that PD individuals with visual hallucinations would have increased hippocampal diffusivity compared to those with uncomplicated PD and healthy individuals.

We also predicted that structural abnormalities of the hippocampus would be accompanied by altered functional connectivity with the rest of brain. Functional connectivity analysis is a relatively novel technique which allows the investigation of large-scale functional networks across the entire brain based on resting-state blood oxygen level dependent (BOLD) signal fluctuations in a very low-frequency range (0.01–0.08 Hz) (Fox and Raichle 2007; Vincent et al. 2007; Zhang and Raichle 2010). Resting-state functional connectivity has identified various intrinsic cortical and cortico-subcortical networks (Fox and Raichle 2007; Robinson et al. 2009; Zhang and Raichle 2010) that are disrupted in Parkinson’s Disease (Baudrexel et al. 2011; Hacker et al. 2012) and in patients with schizophrenia who experience auditory and visual hallucinations (Amad et al. 2013). Here we examined hippocampal functional connectivity in patients with PD and visual hallucinations for the first time.

Visuospatial memory impairment has been reported in those with PD and visual hallucinations (Barnes and Boubert 2011), as well individuals with dementia with Lewy bodies (Hamilton et al. 2008, 2011). Visuospatial memory relies heavily upon the hippocampus (Dupret et al. 2010) and is thought to contribute to visual hallucinations (Barnes and Boubert 2011). Therefore inter-relationship between hippocampal pathology postulated and this critical cognitive faculty was examined (Robbins et al. 1998). Age and cognitive ability may impact on hippocampus (Atienza et al. 2011; Pereira et al. 2013) and were controlled for. We predicted that hippocampal microstructural integrity and functional connectivity would be associated with impaired visuospatial memory performance in people with PD and visual hallucinations.

## Materials and methods

### Subjects

Sixty-six individuals (17 HCs and 49 with PD) were initially recruited to the study as approved by the local hospital Research Ethics Committee.

The participants with PD were diagnosed according to the UK Parkinson’s Disease Society Brain Bank criteria. Clinical assessments included: Hoehn and Yahr Scale (Hoehn and Yahr 1967) to assess stage of illness; Unified Parkinson’s Disease Rating Scale (UPDRS; Fahn and Elton 1987), part III to evaluate PD motor symptom severity; handedness; Montgomery–Åsberg Depression Rating Scale—self-assessment (MADRS-S) (Svanborg and Asberg 1994) to rate depressive symptoms; Mini-mental State Examination (MMSE) (Folstein et al. 1975) to assess cognitive impairment; and the Parkinson Psychosis Rating Scale (PPRS) (Friedberg et al. 1998) which includes a quantitative description of visual hallucinations recorded from patients and caregivers. The score of first item in PPRS was used to measure visual hallucination severity.

The patients with visual hallucinations experienced repetitive and complex visual hallucinations usually of well-formed persons, animals or objects, lasting for at least 4 weeks, and occurring at least once every 4 weeks. Exclusion criteria were: neurological disorders other than Parkinson’s disease; major psychiatric disorders; mild to moderate depressive symptoms (MADRS ≥6); cognitive impairment resulting in a MMSE score <24 (Folstein et al. 1975) to exclude obvious dementia. All subjects were right handed. Thus, 41 individuals were included in this study, and divided into three groups: 12 PD patients with visual hallucinations (PDVH); 15 PD patients without visual hallucinations (PDnonVH); and 14 matched healthy controls (HC).

Patients were described as “on” during the assessment, and their levodopa-equivalent daily dose (LEDD) was calculated according to a standard formula (Krack et al. 1998; Vingerhoets et al. 2002). All subjects were reimbursed for travel expenses, and provided signed informed consent. Demographic and clinical characteristics of the sample are shown in Table 1.

**Table 1:** Demographic and clinical profile of HC, PDnonVH, and PDVH

Demographics	HC (*n* = 14)	PDnonVH (*n* = 15)	PDVH (*n* = 12)	*p* value	*p* value (covaried)
Age^a^	63 (62,68.75)	66 (62,72)	70 (64,72.75)	0.312	n/a
Gender (male/female)	8/6	10/5	10/2	0.35	n/a
Education (years)	7.6 ± 1.4	6.3 ± 1.1	7.2 ± 1.3	0.75	n/a
Duration of illness (years)	n/a	7.1 ± 5.1	9.1 ± 3.5	0.27	n/a
Duration of VH (years)	n/a	n/a	2.4 ± 1.1	n/a	n/a
Hoehn and Yahr stage	n/a	2.9 ± 0.7	3.1 ± 0.7	0.43	n/a
Head motion (mm)	0.093 ± 0.014	0.094 ± 0.021	0.098 ± 0.017	0.98	n/a
Levodopa dose (mg)	n/a	689.7 ± 553.9	986.9 ± 463.8	0.17	n/a
Affected body side (R/B/L)	n/a	6/3/6	5/4/3	0.63	n/a
MMSE score^a^	29 (28,29.25)	29 (28,30)	28.5 (24,29.75)	0.513	n/a
MADRS-S score^a^	0(0,1)	0 (0,2.5)	1 (0.5,3.25)	0.034*	n/a
UPDRS-III score^a^	n/a	17 (14,31)	17.5 (11.25,32.5)	0.719	n/a
Visual hallucinations symptom score	1.0 ± 0.0	1.0 ± 0.0	2.4 ± 0.5	<0.001**	n/a
Hippocampal volume (R)	3,909.8 ± 105.5	3,667.3 ± 151.5	3,847.8 ± 202.4	0.50	0.58
Hippocampal volume (L)	3,695.1 ± 128.5	3,419.5 ± 171.8	3,578.0 ± 114.4	0.39	0.51
Hippocampal MD (R) (10^−3^)	1.004 ± 0.052	0.998 ± 0.051	1.084 ± 0.088	0.002**	0.01**
Hippocampal MD (L) (10^−3^)	0.946 ± 0.040	0.965 ± 0.097	1.032 ± 0.080	0.02*	0.05*
DMS percent correct simultaneous	90.7 ± 10.0	90.0 ± 14.1	80.0 ± 15.6	0.12	n/a
DMS percent correct all delay	76.9 ± 2.8	71.1 ± 3.6	61.0 ± 4.9	0.02*	n/a
PAL total trials adjusted	14.5 ± 5.0	24.8 ± 14.4	25.3 ± 8.7	0.02*	0.05*
PAL first trial memory score	16.7 ± 3.3	12.8 ± 4.7	10.6 ± 4.9	0.006**	0.02*
PAL total errors adjusted	24.8 ± 29.6	66.7 ± 61.0	71.6 ± 46.0	0.04*	0.08
PAL stages completed	7.8 ± 0.4	6.9 ± 1.9	7.2 ± 1.3	0.23	0.40

Continuous data are presented in mean ± SD. *p* values of two group comparisons were calculated using Independent-Samples *t* tests (Chi-squared test for gender and affected body side); *p* values of three group comparisons were calculated using One-way ANOVA

*MMSE* Mini-mental State Examination, *MADRS-S* Montgomery–Åsberg Depression Rating Scale—self-assessment, *UPDRS-III* Unified Parkinson’s Disease Rating Scale, part three, *VH symptom score* the first item score of PPRS, Parkinson Psychosis Rating Scale, *DMS* delayed matching to sample, *PAL* paired associates learning, *PDnonVH* Parkinson’s disease without visual hallucination, *PDVH* Parkinson’s Disease with visual hallucination, *R* right side, *L* left side, *B* both, *n*/*a* not applicable, *p value* (*covaried*) *p* value controlled for age and MMSE

^a^Non-parametric tests, the description is median (percentile 25, percentile 75)

* *p* < 0.05

** *p* < 0.01

### Visuospatial memory and non-spatial memory test

Visuospatial memory was tested using subtests of the Cambridge Neuropsychological Test Automated Battery (CANTAB; Cambridge Cognition Ltd) (Robbins et al. 1998): Paired Associates Learning (PAL). Two patients with PDVH did not carry out the PAL test. Since PDVH has been linked to impaired visual accuracy (Matsui et al. 2006), which can confound interpretation of visuospatial memory performance, the DMS (Delayed Matching to Sample) test in ‘spontaneous exhibition mode’ (without any memory load) was used to provide a baseline measure of visual accuracy in participants and controlled in PAL analyses. The DMS test was run in ‘delay exhibition mode’ and was used to provide a measure of non-spatial working memory.

### MRI data acquisition

MRI scans were carried out on a 3.0 T Philips MRI scanner. Three-dimensional T1-weighted anatomical MRI were acquired with fast field echo sequence (Magnetization Prepared Rapid Gradient Echo, MPRAGE), with the following parameters: TR = 6,895 ms; TE = 3.16 ms; FA = 8°; 1 × 1 × 1 mm^3^ voxels; FOV = 250 × 250 × 155 mm^3^; number of slices = 155.

Diffusion-weighted images were collected using a single-shot spin-echo imaging sequence with a voxel size of 2 × 2×2 mm^3^ with the following parameters: FOV = 225 × 180 × 225 mm^3^, acquisition matrix = 112 × 112; TR = 11,948 ms; TE = 65 ms; flip angle = 90°; slice thickness = 2 mm; number of slices = 90. Diffusion sensitizing gradients (*b* = 1,000 s/mm^2^) were applied in 16 directions. One additional image was collected without diffusion gradient (*b*_0_ = 0 s/mm^2^). Scanning was repeated twice to increase the signal/noise ratio.

The functional imaging data were acquired using a gradient-echo echo-planar imaging (EPI) sequence sensitive to BOLD contrast (Kwong et al. 1992; Ogawa et al. 1992). Acquisition parameters were: TR = 1,800 ms; TE = 30 ms; flip angle = 90°; 220 volumes; 45 contiguous axial slices; anterior–posterior acquisition; in-plane resolution = 3.75 × 3.75 mm^2^; slice thickness = 4 mm; field of view = 240 × 180 × 240 mm^3^; acquisition time = 6.6 min. Slice acquisition order was contiguous. Earplugs were used to reduce scanner noise and head motion was restricted by a foam pillow, as well as extendable padded head clamps. Participants were asked to simply rest in the scanner with their eyes closed before each resting-state scan and not fall asleep while remaining as still as possible.

### Image preprocessing

T1-weighted structural data were processed with the VBM8 toolbox (http://dbm.neuro.uni-jena.de/vbm/) in SPM8 (http://www.fil.ion.ucl.ac.uk/) using Matlab (Matlab 7, The MathWorks, Natick, MA, USA). T1-weighted images were bias-corrected and segmented into gray matter (GM), white matter (WM), and CSF, and then affine normalized to MNI space. Tissue class images were then non-linearly normalized using the high-dimensional DARTEL (Diffeomorphic Anatomical Registration Through Exponentiated Lie Algebra) algorithm to study-specific templates created by DARTEL. The transformation matrix was then applied to T1-aligned MD, resting fMRI maps and original segmented hippocampal structures (see below).

Mean modulated and smoothed gray matter maps (gray matter intensity threshold = 0.2) were used to generate a group gray matter mask and applied as a mask for analyzing functional connectivity differences in between-group comparisons, specific to the groups involved in a particular test.

DTI data were pre-processed and analyzed using FMRIB Software Library, version 4.1 (FSL; http://www.fmrib.ox.ac.uk/fsl). Diffusion-weighted images were converted from dicom to nifti format, and corrected for eddy currents and subject motion by affine registration to the first b0 image using the FSL “eddy_correct” function. Diffusion tensors were linearly fitted to the diffusion-weighted images using the FSL tool “dtifit” to generate an MD output map.

Resting fMRI data preprocessing was performed using SPM8 (http://www.fil.ion.ucl.ac.uk/spm). First, all functional images were corrected for slice timing as well as head movement. Participants with excessive head movement >2.5 mm of displacement or >2.5° of rotation in any direction were discarded (four PDnonVH patients and two PDVH patients). Prior to band-pass filtering (0.01–0.08 Hz) which controls for the physiological “noise”(cardiac and respiratory-related artifacts), the following nuisance covariates were regressed from the BOLD signal (Weissenbacher et al. 2009): six rigid-body parameters; white matter signal; cerebrospinal fluid signal.

### Measurement of head motion

As described by others (Van Dijk et al. 2012; Satterthwaite et al. 2012), our measurement of head motion was mean motion, which represents the mean absolute volume-to-volume displacement of each brain estimated from the translation parameters in the *x* (left/right), *y* (anterior/posterior), and *z* (superior/inferior) directions [displacement = square root (*x* ^2^ + *y* ^2^ + *z* ^2^)] and expressed in mm. Given that movement has an impact on ALFF (Satterthwaite et al. 2012), and mean motion linearly correlates with ALFF in diverse brain regions, the mean motion parameter was estimated and compared in both analyses.

### Hippocampal volume and mean MD

To register DTI data to the T1-weighted images, we calculated a full affine transformation from FA maps to brain-extracted whole-brain volumes of T1-weighted images (Carlesimo et al. 2012) using FLIRT (Jenkinson and Smith 2001; Jenkinson et al. 2002). The transformation matrix was then applied to the MD maps. Bilateral hippocampus of anatomical T1-weighted images were then automatically segmented using FIRST (Patenaude et al. 2011) by running a two-stage affine registration to MNI152 space. Segmented structures were used as binary masks for which mean MD was calculated from realigned MD map for each individual.

### Vertex analysis

FIRST was used to investigate localized shape differences in the hippocampus. A surface mesh was created for each hippocampus using a deformable mesh model, and aligned to a common space prior to investigating group differences. The mean surface from the FIRST models (in MNI152 space) was used as the target to which surfaces from each individual were aligned. Vertex analysis was performed using first utils (Patenaude et al. 2011) and hippocampal shape difference was assessed on a per vertex basis. Regional differences in the vertices across the three groups were assessed using a global linear model. The results were corrected for multiple comparisons using a family-wise error (family-wise error, *p* < 0.05) correction.

### Voxel-based MD in hippocampal regions

MD maps were normalized to the Montreal Neurological Institute (MNI) space by applying the transformation parameters obtained from the structural images. A normalized hippocampal mask was applied in each group comparison for statistical analysis of hippocampal MD.

### Functional Connectivity of hippocampus

Functional connectivity was based on a seed-region approach and conducted in MNI space. For each participant, the mean time series was extracted from bilateral hippocampal regions defined by fMRI-realigned hippocampus, and correlated with the appropriate time series at all other brain voxels in the original acquisition space using Pearson’s coefficient of correlation followed by Fisher’s *r*- to -*z* transformation. Functional connectivity maps were then normalized to the Montreal Neurological Institute (MNI) space by applying the transformation parameters obtained from the structural images to those time and motion corrected and nuisance covaried images, resampled (3 × 3 × 3 voxels) and smoothed (4-mm full width at half maximum (FWHM) Gaussian kernel).

### Statistical analysis

Between-group voxel-wise comparisons were performed using two sample t test. Voxel-by-voxel statistical analysis of MD differences between PDVH, PDnonVH and healthy controls was restricted to within the hippocampal mask, and the results were corrected for multiple comparisons (family-wise error) using a threshold of *p* < 0.05. The functional connectivity statistical comparisons were restricted within the corresponding gray matter mask. The statistical tests for functional connectivity were corrected for multiple comparisons to a significance level of *p* < 0.05 using Monte Carlo simulations (uncorrected single voxel significance level of *p* < 0.05 and a minimum cluster size based on the size of the gray matter mask of each load of comparison) (Ledberg et al. 1998). Age and MMSE score were introduced as nuisance covariates in all statistical analyses.

One-way ANOVA was used to compare demographic factors, visual-memory scores, hippocampal MD and volume using Statistical Package for Social Sciences (version 15.0.1) (SPSS for Windows 2006). Post hoc tests were carried out where appropriate. Mean hippocampal functional connectivity *z* scores were calculated for each cluster which had a difference between PDVH and PDnonVH, and Pearson correlation co-efficients between hippocampal MD, mean functional connectivity *z* scores, visual hallucinations severity and visuospatial memory score were calculated in PDVH group. Age and MMSE scores were controlled for within-group comparisons of hippocampal volume and MD, and intracranial volume was additionally controlled in group comparisons of hippocampal volume. Age and visual accuracy (DMS score) were controlled in PAL between-group comparisons and correlational analyses. The relationship between levodopa-equivalent dosage and bilateral hippocampal MD/functional connectivity results in PD patients was assessed with Pearson correlation analysis. A similar analysis between PD severity (UPDRS-III score) and bilateral hippocampal MD/functional connectivity results in the PD group was also undertaken. Pearson Chi-square tests between gender and hippocampal MD/functional connectivity results in the PD group were carried out as well.

## Results

### Demographic and clinical profile

Clinical data comparisons are summarized in Table 1. Participants were matched for age (>55 years), gender, education, and MMSE score. The PD groups were also balanced for duration of illness, drug dosage, and PD symptom severity. In PDVH group, five reported having visual hallucinations about 1–3 times per month, five had visual hallucinations 1–2 times per week, and two reported having visual hallucinations every day. Nine patients experienced visual hallucinations of people, 1 subject of animal and objects, one of only objects, and one patient experienced “presence of a person” hallucinations. Three patients with visual hallucinations were under antipsychotic drug quetiapine. Two patients reported visual illusions in addition to visual hallucinations. No patient experienced delusion or delirium. PD patients were receiving levodopa medication. Participants were asked if they experienced hallucinations in the scanner—none were reported.

### No group differences in visual accuracy

The three groups did not differ significantly in visual accuracy. This parameter was included as a ‘nuisance’ covariate for later correlation and group comparison analyses of performance in the visuospatial memory test (PAL).

### Visuospatial memory was impaired in PD

PD groups had significantly poorer performance than controls on PAL indices. Post hoc tests (see Supplementary Table 1; Supplementary Fig. 1) confirmed both PDVH and PDnonVH groups performed worse than the HC group and there was no significant difference between PDVH and PDnonVH.

### Hippocampal MD was increased in PDVH

There were no group differences in overall hippocampal volume and shape, but PDVH had higher MD values in the right hippocampus compared to PDnonVH and HC groups, and greater MD in left hippocampus compared to HC.

**Fig. 1:**
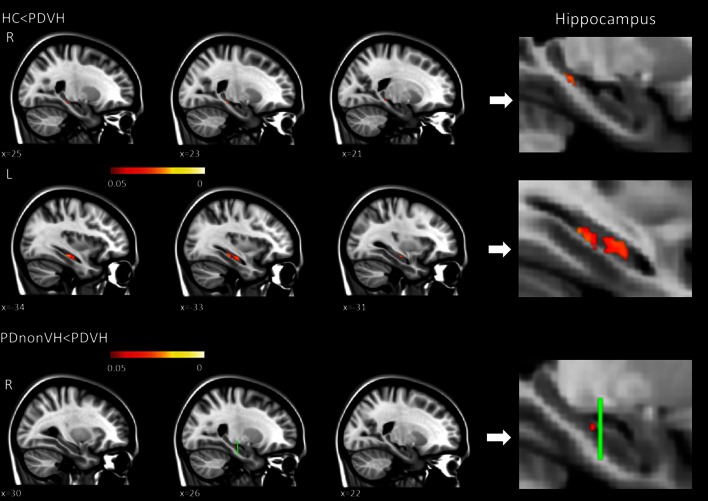
Group differences in hippocampal MD. Voxel-by-voxel analyses of increased hippocampal diffusivity in patients with Parkinson disease with visual hallucinations compared with healthy controls and PD individuals without visual hallucinations. Statistical results are superimposed on the standard Montreal Neurological Institute template (*x* coordinates are reported). Age and MMSE were controlled. The *green line* in the *bottom panel* shows the middle plane (*y* = −22 mm) of the right hippocampal mask generated in this study

In voxel-by-voxel analyses, the PDVH group had greater MD in right posterior hippocampal regions compared to PDnonVH and HC groups and greater MD in left hippocampal body compared to HC. There was no correlation between visual spatial memory score and hippocampal MD in PDVH group. There was no significant correlation between levodopa-equivalent dosage/gender and bilateral hippocampal MD results in PD patients, and no significant correlation between PD severity and hippocampal results in PD patients (Fig. 1).

### The severity of visual hallucinations is linked with impaired visual-memory performance

The severity of visual hallucinations indexed by PPRS question 1 was correlated with visuospatial memory performance in PDVH group. The correlation coefficients with visual hallucinations severity were: PAL Total trials adjusted *r* = 0.694, *p* < 0.05; PAL First trial memory *r* = −0.686, *p* < 0.05; PAL Total errors adjusted *r* = 0.843, *p* < 0.01; PAL Stages completed *r* = −0.725, *p* < 0.05.

### Functional connectivity

**Fig. 2:**
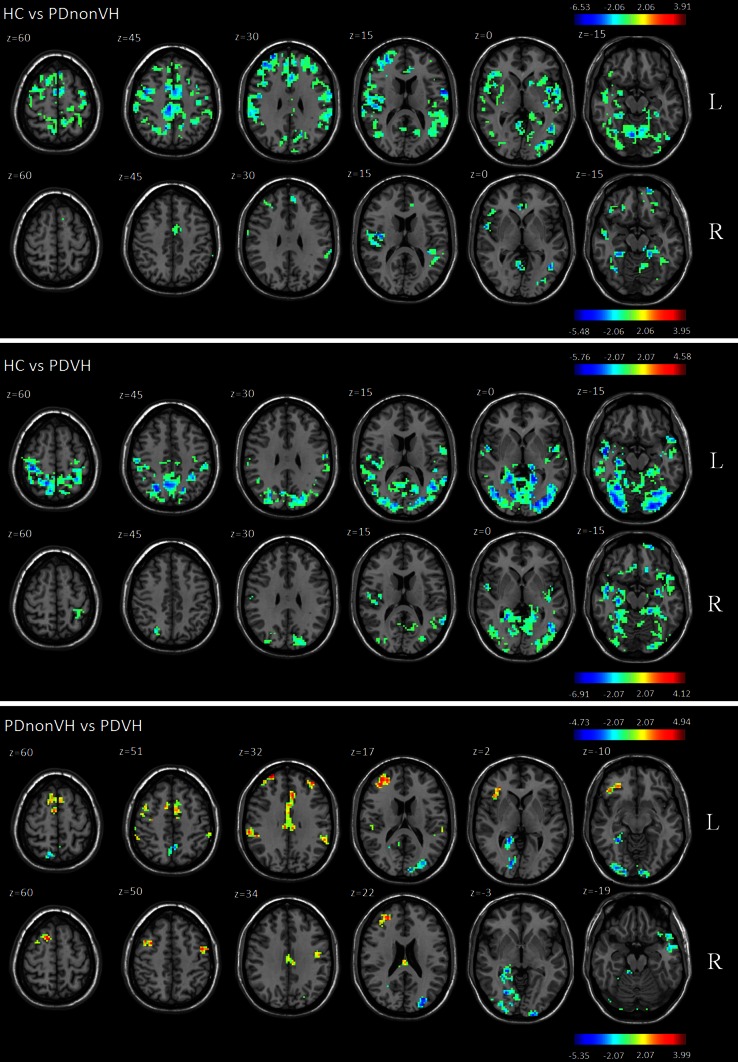
Group differences in hippocampal functional connectivity. Statistical results are superimposed on the standard Montreal Neurological Institute template (*x* coordinates are reported) (mm) at the given threshold corrected for multiple comparisons [Monte-Carlo Simulation, cluster size = 1,701 mm^3^ (63 voxels), in comparison between HC and PDnonVH, *T* > 2.06 (or *T* < −2.06) in comparison between HC and PDVH, and in comparison between PDnonVH and PDVH, *T* > 2.07 (or *T* < −2.07)]. Age and MMSE scores are entered as covariables. In *each panel*, *blue* indicates relatively lower functional connectivity in the second group compared to the first; *red* indicates the reverse. The *right side* of the figure is the left side of the brain. *L* functional connectivity with left hippocampus, *R* functional connectivity with the right hippocampus

Both PD groups had lower hippocampal functional connectivity across widespread cortical areas compared to HC. In the PDnonVH group, lower hippocampal functional connectivity was mainly located in parieto-frontal regions, whereas in PDVH group, lower hippocampal functional connectivity was mainly located in occipito-parietal and temporal regions (Fig. 2).

Compared to PDnonVH, PDVH had lower functional connectivity between right hippocampus and bilateral cuneus and lingual gyrus, right fusiform gyrus, right medial temporal lobe and left superior/middle temporal gyrus, as well as lower FC between left hippocampus and bilateral lingual gyrus, right fusiform gyrus, left cuneus, right medial temporal lobe and right precuneus. PDVH also had greater hippocampal FC in bilateral frontal lobe, cingulate cortex, and inferior parietal lobe which are components of default mode network and salience network (Fig. 2; Table 2).

**Table 2:** Group differences between PDnonVH and PDVH in hippocampal functional connectivity

FC	Number of voxels	*x*	*y*	*z*	Side	Brain regions	BA	*T* value
Left hippocampus								
Lower in PDVH	108	0	−96	−18	L	Occipital lobe/lingual gyrus	18/17	−3.4775
	227	33	−90	−12	R	Inferior occipital gyrus/lingual gyrus/fusiform gyrus	18/17	−4.7271
	165	18	−51	9	R	Medial temporal lobe/parahippocampa gyrus/precuneus/fusiform gyrus/lingual gyrus	37/19	−3.476
	73	−9	−93	18	L	Middle occipital gyrus/cuneus	19/18	−3.5145
	73	0	−57	51	L/R	Parietal lobe/precuneus	7	−3.3123
Higher in PDVH	146	42	24	6	R	Inferior frontal gyrus/middle frontal gyrus/insula/inferior orbital frontal gyrus	11/13	3.8879
	196	27	57	30	R	Middle frontal gyrus/superior frontal gyrus	10	4.4842
	161	63	−33	45	R	Inferior parietal lobe/postcentral gyrus	40	3.5631
	555	−6	18	39	R/L	Superior frontal lobe/anterior cingulate gyrus/medial temporal lobe/medial frontal gyrus/	24/32/6/23	4.5253
	95	−39	24	24	L	Middle frontal gyrus/inferior frontal gyrus/superior frontal gyrus	9	3.7289
	96	−63	−39	45	L	Inferior parietal lobe/supramarginal gyrus/superior temporal gyrus	40	3.3531
	92	45	3	54	R	Middle frontal lobe/Precentral gyrus	6	3.3516
Right hippocampus								
Lower in PDVH	103	−36	21	−24	L	Superior temporal gyrus/middle temporal gyrus/	38	−3.6082
	156	33	−90	−12	R	Inferior occipital gyrus/lingual gyrus/middle occipital gyrus	18	−4.709
	117	−24	−105	−6	L	Inferior occipital gyrus/lingual gyrus/cuneus	18	−4.3514
	379	27	−48	−6	R	Medial temporal lobe/lingual gyrus/fusiform gyrus/parahippocampal gyrus/cuneus/cerebellum	19/30/17/18	−4.7061
	105	−27	−81	21	L	Middle occipital gyrus/cuneus/superior occipital lobe	19/18	−3.8056
Higher in PDVH	108	27	45	24	R	Middle frontal gyrus/superior frontal gyrus	10	3.5069
	67	−3	−24	24	L	Posterior cingulate cortex/medial temporal lobe	23	3.9886
	71	−48	−3	48	L	Frontal lobe/precentral gyrus	6/4	3.7758
	94	36	9	45	R	Middle frontal gyrus/superior frontal gyrus	6	3.6509

Group differences in functional connectivity are shown at *p* < 0.05 corrected for multiple comparisons [cluster size = 1,701 mm^3^, *T* > 2.07 (or *T* < −2.07)]. *x*, *y*, *z* coordinates in the MNI atlas extend from *z* = −60 to *z* = +85 mm. *T* values are from a *t* test of the peak voxel (showing greatest statistical difference within a cluster), and a negative *T* value means lower ALFF in PDVH group. Age and MMSE scores are regressed out as nuisance variables

*BA* Brodmann area, *L*, *R* left and right

**Fig. 3:**
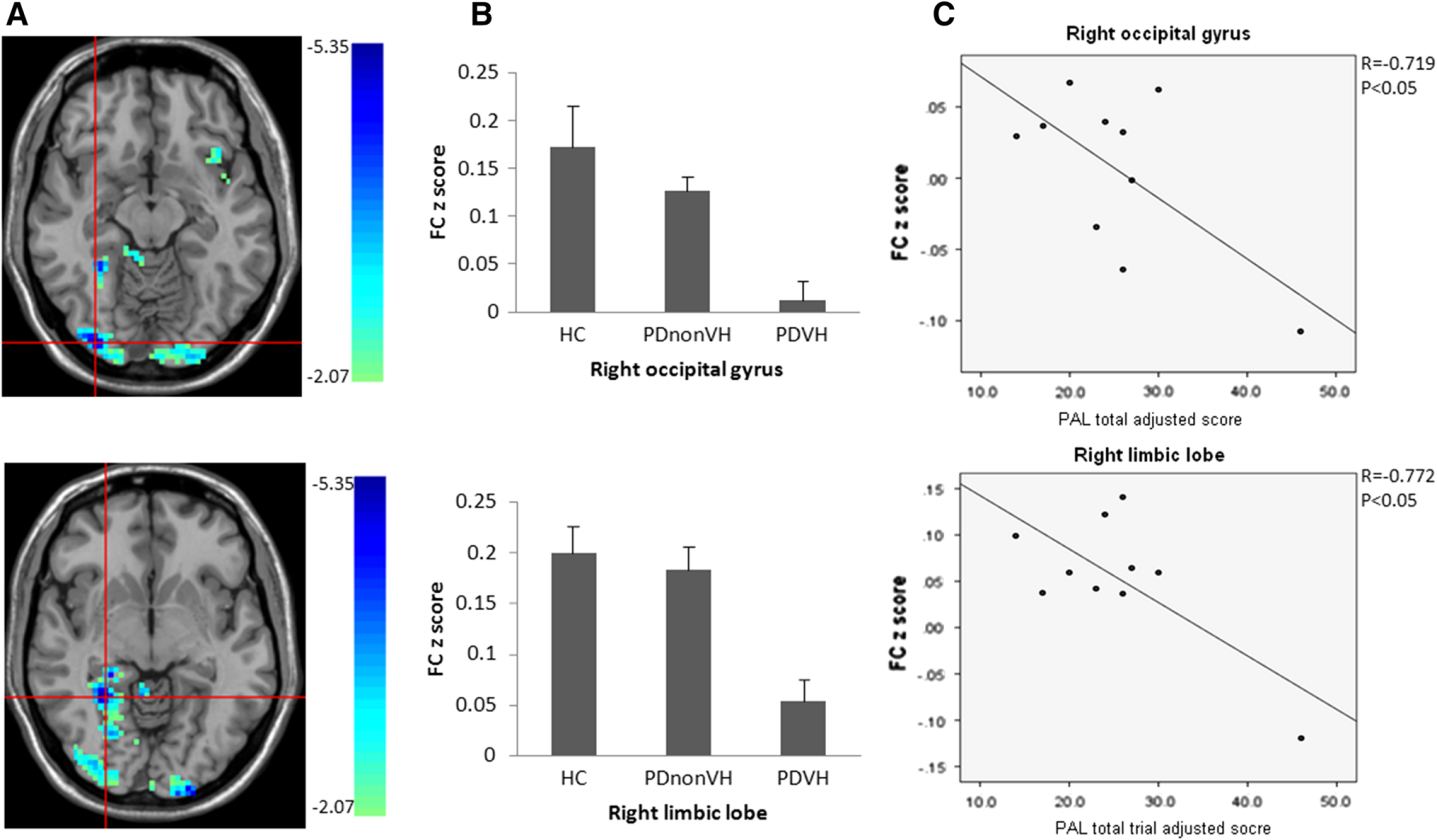
Correlation analyses of hippocampal functional connectivity and visuospatial memory performance. **a** T map of the two clusters showing significant correlation between functional connectivity and visuospatial memory score in PDVH patients. MNI coordinates (*x*, *y*, *z*): *top right* occipital gyrus = 33, −90, −12; *bottom right* medial temporal lobe = 27, −48, −6 (only clusters with decreased functional connectivity in PDVH are shown here). **b** *Bar graphs* of the region of interest (ROI)—average functional connectivity *z* scores (±standard error) for patient groups and HC group in right occipital gyrus (HCs 0.172; PDnonVH 0.126; PDVH 0.012; *p* < 0.001) and right medial temporal lobe (HCs 0.199; PDnonVH 0.183; PDVH 0.053; *p* < 0.001). **c** Correlation between hippocampal functional connectivity *z* score and visuospatial memory performance (PAL scores) in PDVH group. Scatterplot of ROI-averaged functional connectivity *z* scores from occipital cluster (with total trial adjusted score: *r* = 0.719, *p* < 0.05) and medial temporal cluster against visuospatial memory scores (with total trial adjusted score: *r* = 0.772, *p* < 0.05). Correlation coefficients were controlled for age and visual accuracy scores

Moreover, we carried out a correlation study between the mean functional connectivity *z* scores in the clusters with significant difference between PDVH and PDnonVH and cognitive tests (see Supplementary Table 2). The results showed that PDVH group has lower right hippocampal functional connectivity with right occipital gyrus (peak voxel of the cluster: *x* = 33, *y* = −90, *z* = −12) and right medial temporal regions (peak voxel of the cluster: *x* = 27, *y* = −48, *z* = −6), and the mean functional connectivity *z* scores in the two clusters were associated with worse visuospatial memory performance (Fig. 3).

There was no significant correlation between levodopa-equivalent dosage/gender and functional connectivity in PD (please see bottom panel of Fig. 2), and no significant correlation between PD severity and functional connectivity in patients.

## Discussion

The current study, for the first time, reports the microstructural and functional connectivity differences in the hippocampus in PD patients with visual hallucinations, and it correlates with the extent to which visuospatial memory is impaired with hippocampal pathology and visual hallucination severity. Our study thus consolidates a pivotal role of hippocampus in the production of visual hallucinations in PD, similar to its reported role in other psychosis such as schizophrenia (Amad et al. 2013).

### Hippocampal microstructure

The results are consistent with our hypothesis that hippocampal microstructure is altered in individuals with PD and visual hallucinations compared to both PDnonVH and HC. The patients with visual hallucinations had higher MD in right hippocampus relative to both PDnonVH and HC groups, and higher MD in left hippocampus relative to HCs. The results remained significant when possible confounds due to age (Lim et al. 1990; Driscoll and Sutherland 2005) and cognitive ability were controlled (using the MMSE and DMS measure of non-spatial working memory).

Our results are partly in line with previous findings of hippocampal gray matter deficits in PD hallucinators (Ibarretxe-Bilbao et al. 2008) in which a whole-brain voxel-based analysis found no significant gray matter intensity difference between PDVH and PDnonVH. Ibarretxe-Bilbao and colleagues did report a hippocampal ‘volume’ deficit in PDVH patients compared to HCs, whereas we found no hippocampal volume differences between groups. Our result is, however, consistent with a recent study of whole-brain macrostructural differences in PD, which also observed no volume alterations in the hippocampus (Gama et al. 2014).

Although the neural mechanism of abnormal diffusivity in gray matter structures is not fully understood, it is generally assumed that, in pathologic states, greater diffusivity signals more water content or disruption and break-down of tissue cytoarchitecture (den Heijer et al. 2012). For example, progressive loss of cellular barriers or an increase in extracellular water space in neurodegeneration could lead to an increase in MD (Basser and Pierpaoli 1996; den Heijer et al. 2012), and reflect a deterioration in the efficacy of synaptic and extrasynaptic transmission (Sykova and Nicholson 2008). Resonating with this idea, increased gray matter MD has been linked to impaired cognitive performance in mild cognitive impairment or Alzheimer’s disease (Muller et al. 2007; Cherubini et al. 2010). Together with the absence of group differences in hippocampal volume and shape observed here, normal neuron counts in the hippocampus in PD, but increased Lewy bodies in the hippocampal regions of those with PDVH (Churchyard and Lees 1997; Kalaitzakis et al. 2009; Gallagher et al. 2011), one possibility is that that increased hippocampal diffusivity in patients with visual hallucinations may be due to disruption caused by α-synuclein deposition in neurons. An alternative explanation is that, because MD is sensitive to water content, and hence to CSF around the hippocampus, greater MD could be a ‘partial volume’ effect, due to a smaller hippocampus size (and hence more CSF in the DTI voxels). However, although we observed a trend level difference in hippocampal size across the groups in this study (*p* = 0.08), compared to controls, the hippocampus was actually smaller in the PDnonVH group, but gray matter MD was not increased in this group. This suggests that volume and gray matter MD are relatively independent indices, and the increase in hippocampal MD in PDVH cannot easily be explained by a decrease in volume.

Significant MD alteration was localized to the posterior right hippocampus in the PDVH group compared to both PDnonVH and HC. In primates, the posterior portions of the hippocampus correspond to the rodent dorsal hippocampus, which is needed for visuospatial memory processing, while the ventral (anterior) part of hippocampus is involved in non-spatial memory and emotional behavior (Behrendt 2010; Fanselow and Dong 2010). Greater diffusivity in right posterior hippocampus and impaired spatial memory performance in PDVH is consistent with a possible mediating role for impaired visuospatial memory in the generation of VH; indeed, visuospatial memory impairment was correlated with the severity of visual hallucinations.

### Hippocampal functional connectivity

The segmented hippocampus was used as seed region of interest to investigate functional connectivity with the rest of the brain. Compared with the PDnonVH group, PD hallucinators had lower functional connectivity between hippocampus and cuneus, lingual gyrus, fusiform gyrus, medial temporal lobe, superior/middle temporal gyrus, and precuneus. The parietal regions belong to the dorsal visual pathway, whereas the temporal regions belong to the ventral visual pathway. Both pathways originate in occipital regions then project through inferior parietal and inferior temporal gyri respectively to the perirhinal-hippocampal cortices (Behrendt 2010). The latter is critical for complex visual processing including memory, image perception, and internal expectations (Downing et al. 2006; Rolls and Xiang 2006; Bussey and Saksida 2007; Cerf et al. 2010), as well as contextual and scene representation (Chen et al. 2012; Hebart and Hesselmann 2012) and visuospatial attention (Nassi and Callaway 2009). Thus, the hippocampus is in a unique position for the conjunction of stimulus-related and spatial and non-spatial contextual information processed in parietal (dorsal) and temporal (ventral) regions (Behrendt 2010). Our results fit well with this notion that integration of the dorsal and ventral visual streams in the hippocampus is disrupted in patients with visual hallucinations. They are also consistent with previous studies, suggesting that disorganization of neuronal spikes in hippocampus might disrupt integration of ventral/dorsal information and induce visual hallucination rather than reflect reality (Olypher et al. 2006).

The patients with visual hallucinations had greater functional connectivity between the hippocampus and regional components of the default mode network (DMN) (including medial frontal gyrus, posterior cingulate cortex (PCC) and bilateral inferior parietal gyrus) and ‘salience’ network [composed of anterior insula and anterior cingulate gyrus (ACC)]. This extends our previous work showing that this cohort of PD patients with visual hallucinations have greater connectivity within the DMN itself (Yao et al. 2014).

The DMN processes spontaneous thoughts, consciousness, memory, and social cognition in resting state (Raichle et al. 2001; Greicius et al. 2003; Buckner et al. 2008), and is known to be functionally and structurally connected with hippocampal and parahippocampal regions (Buckner et al. 2008; Ward et al. 2013). The salience network is also involved in integration and “harmonization” of external sensory stimuli and internal states (Seeley et al. 2007), (Sridharan et al. 2008). Thus abnormalities in hippocampus/DMN/salience network connectivity fits with theories that visual hallucinations result from limited “bottom-up” peripheral sensory input from primary visual cortex and unconstrained “top-down” processing of endogenous memories and attention from frontal regions (Bar et al. 2006; Behrendt 2010; Onofrj et al. 2012).

### Hippocampal functional connectivity correlates with visuospatial memory performance

We found a correlation between functional connectivity and visual–spatial memory in PDVH individuals. Moreover, visual hallucinations severity was associated with visuospatial memory in patients with visual hallucinations. These results support the notion that both disrupted intrinsic integration with attenuated extrinsic visual stimuli from primary visual cortex contribute to reduced visuospatial memory performance, thus might underlie visual hallucinations in PD.

To sum up, the present multimodal MRI techniques provided reliable and novel results. The microstructural and functional connectivity findings are consistent with the hypothesis of imbalanced integration of internal memory and external visual input (Diederich et al. 2005; Goetz 2009) which contribute to visual hallucinations in Parkinson’s disease, and implicating that this pathological phenomenon can be underlied by the microstructural deficit and dysfunction in hippocampus. The consistent result of the hippocampal biomarker indicated in the present study supports future development of therapeutic strategy on the basis of neuro-guided stimulation approach, such as transcranial magnetic stimulation (TMS) (Liu et al. 2013) and transcranial direct current stimulation (tDCS) (Brunoni et al. 2013) to modulate the connectivity strength of visual networks.

### Limitations

We acknowledge that our study had a relatively modest sample size. However, given the practical difficulty in recruiting and scanning this population, our numbers compare favorably to the existing literature. To compensate for the limited sample size, we pursued a multimodal approach which provided highly convergent findings supporting hippocampal pathology and visuospatial memory impairment in PD patients with visual hallucinations.

The levodopa-equivalent dosage is not perfectly matched in our PD patients with and without visual hallucinations, and this might contribute to result bias. The side-effects of pro-dopaminergic medication were originally assumed to be the most important causal factor for VH in PD (Moskovitz et al. 1978), however, more recent evidence suggests VH is an intrinsic part of PD (Sanchez-Ramos et al. 1996; Fenelon et al. 2000; Merims et al. 2004). Moreover, when we controlled levodopa-equivalent dosage in our analysis, the result did not change (see Supplementary Fig. 2).

We also acknowledge the MMSE used in our study is a rather insensitive instrument for measurement of memory function and memory impairment is common in non-demented patients with PD (Yarnall et al. 2014). However, when the levodopa**-**equivalent dose, non-spatial memory performance and head movement were included as covariates the pattern of results did not alter (please see Supplementary Fig. 2). Moreover, three patients with hallucinations also received antipsychotic medication, and while it is possible that our results could potentially be influenced by this treatment, if anything, this should minimize the relationship between hallucination severity and impaired visuospatial memory observed here.

The cross-sectional nature of our study prevents us from drawing causal conclusions. Although we suggest a link between low hippocampal diffusivity and low hippocampal functional connectivity, poor visuospatial memory performance, and visual hallucinations in PD, it may be that visuospatial ability and visual hallucinations are simply comorbid symptoms induced by hippocampal deterioration and dysfunction. Future studies with larger sample sizes will help excavate causal evidence for visual hallucinations in PD.

Finally, there are several other potential methodological limitations to the interpretation of the data. First, we could not completely avoid the effects of physiological ‘noise’ during resting fMRI scans (such as cardiac and respiratory pulsation), that can feature in the resting-state low-frequency rage (0.01–0.08 Hz). However, a low-pass filtering at a cut-off of 0.08 Hz was used in our study to control for physiological “noise” (Fox and Raichle 2007). Second, the patients with visual hallucinations recruited in this study had no obvious depression or dementia, which is quite rare in people with PD and hallucinations (Fenelon and Alves 2010), so we are cautious in generalizing our findings more severely affected individuals.

## Conclusions

The innovative multimodal imaging approach in the current study revealed a microstructural deficit in right hippocampal region in PD patients with visual hallucinations compared to PD non-hallucinators. This impairment was associated with altered functional connectivity in hippocampus across regions involved in integrative processing of visual information, and with impaired visuospatial memory processing in visual hallucinations. Since visual hallucinations herald a poor prognosis in Parkinson’s disesase, these findings may pave the way for future studies of imaging biomarkers or tools to measure treatment response in those most at risk.

## Electronic supplementary material

Below is the link to the electronic supplementary material.

Supplementary material 1 (DOC 30 kb)

Supplementary material 2 (DOC 27 kb)

Supplementary Figure 1 PAL scores of HC, PDnonVH and PDVH groups. Supplementary material 3 (TIFF 1,966 kb)

Supplementary Figure 2 Group differences between PDnonVH and PDVH in hippocampal functional connectivity. Statistical results are superimposed on the standard Montreal Neurological Institute template (x coordinates are reported) (mm) at the given threshold corrected for multiple comparisons (Monte-Carlo Simulation, cluster size=1701 mm^3^ (63 voxels), in comparison between PDnonVH and PDVH, T>2.09 (or T<−2.09)). Age, MMSE scores, levodopa-equivalent dosage, head motion and working memory scores were entered as covariables. In each panel, blue indicates relatively lower functional connectivity in PDVH compared to PDnonVH; red indicates the reverse. The right side of the figure is the left side of the brain. L = functional connectivity with left hippocampus; R = functional connectivity with the right hippocampus. Supplementary material 4 (TIFF 2,027 kb)

## References

[CR1] Aarsland D, Bronnick K, Ehrt U, De Deyn PP, Tekin S, Emre M, Cummings JL (2007). Neuropsychiatric symptoms in patients with Parkinson’s disease and dementia: frequency, profile and associated care giver stress. J Neurol Neurosurg Psychiatry.

[CR2] Amad A, Cachia A, Gorwood P, Pins D, Delmaire C, Rolland B, Mondino M, Thomas P, Jardri R (2013) The multimodal connectivity of the hippocampal complex in auditory and visual hallucinations. Mol Psychiatry 10.1038/mp.2012.181 23318999

[CR3] Atienza M, Atalaia-Silva KC, Gonzalez-Escamilla G, Gil-Neciga E, Suarez-Gonzalez A, Cantero JL (2011). Associative memory deficits in mild cognitive impairment: the role of hippocampal formation. Neuroimage.

[CR4] Bar M, Kassam KS, Ghuman AS, Boshyan J, Schmid AM, Dale AM, Hamalainen MS, Marinkovic K, Schacter DL, Rosen BR, Halgren E (2006). Top-down facilitation of visual recognition. Proc Natl Acad Sci USA.

[CR5] Barnes J, Boubert L (2011). Visual memory errors in Parkinson’s disease patient with visual hallucinations. Int J Neurosci.

[CR6] Basser PJ, Pierpaoli C (1996). Microstructural and physiological features of tissues elucidated by quantitative-diffusion-tensor MRI. J Magn Reson B.

[CR7] Baudrexel S, Witte T, Seifried C, von Wegner F, Beissner F, Klein JC, Steinmetz H, Deichmann R, Roeper J, Hilker R (2011). Resting state fMRI reveals increased subthalamic nucleus-motor cortex connectivity in Parkinson’s disease. Neuroimage.

[CR8] Behrendt RP (2010). Contribution of hippocampal region CA3 to consciousness and schizophrenic hallucinations. Neurosci Biobehav Rev.

[CR9] Bernal-Pacheco O, Limotai N, Go CL, Fernandez HH (2012). Nonmotor manifestations in Parkinson disease. Neurologist.

[CR10] Brunoni AR, Valiengo L, Baccaro A, Zanao TA, de Oliveira JF, Goulart A, Boggio PS, Lotufo PA, Bensenor IM, Fregni F (2013). The sertraline vs. electrical current therapy for treating depression clinical study: results from a factorial, randomized, controlled trial. JAMA Psychiatry.

[CR11] Buckner RL, Andrews-Hanna JR, Schacter DL (2008). The brain’s default network: anatomy, function, and relevance to disease. Ann N Y Acad Sci.

[CR12] Bussey TJ, Saksida LM (2007). Memory, perception, and the ventral visual-perirhinal-hippocampal stream: thinking outside of the boxes. Hippocampus.

[CR13] Carlesimo GA, Piras F, Assogna F, Pontieri FE, Caltagirone C, Spalletta G (2012). Hippocampal abnormalities and memory deficits in Parkinson disease: a multimodal imaging study. Neurology.

[CR14] Cerf M, Thiruvengadam N, Mormann F, Kraskov A, Quiroga RQ, Koch C, Fried I (2010). On-line, voluntary control of human temporal lobe neurons. Nature.

[CR15] Chen Q, Weidner R, Weiss PH, Marshall JC, Fink GR (2012). Neural interaction between spatial domain and spatial reference frame in parietal-occipital junction. J Cogn Neurosci.

[CR16] Cherubini A, Peran P, Spoletini I, Di Paola M, Di Iulio F, Hagberg GE, Sancesario G, Gianni W, Bossu P, Caltagirone C, Sabatini U, Spalletta G (2010). Combined volumetry and DTI in subcortical structures of mild cognitive impairment and Alzheimer’s disease patients. J Alzheimers Dis.

[CR17] Churchyard A, Lees AJ (1997). The relationship between dementia and direct involvement of the hippocampus and amygdala in Parkinson’s disease. Neurology.

[CR18] den Heijer T, der Lijn F, Vernooij MW, de Groot M, Koudstaal PJ, der Lugt A, Krestin GP, Hofman A, Niessen WJ, Breteler MM (2012). Structural and diffusion MRI measures of the hippocampus and memory performance. Neuroimage.

[CR19] Diederich NJ, Goetz CG, Stebbins GT (2005). Repeated visual hallucinations in Parkinson’s disease as disturbed external/internal perceptions: focused review and a new integrative model. Mov Disord.

[CR20] Downing PE, Chan AW, Peelen MV, Dodds CM, Kanwisher N (2006). Domain specificity in visual cortex. Cereb Cortex.

[CR21] Driscoll I, Sutherland RJ (2005). The aging hippocampus: navigating between rat and human experiments. Rev Neurosci.

[CR22] Dupret D, O’Neill J, Pleydell-Bouverie B, Csicsvari J (2010). The reorganization and reactivation of hippocampal maps predict spatial memory performance. Nat Neurosci.

[CR23] Fahn S, Elton R (1987) Members of the updrs development committee. In: Fahn S, Marsden CD, Calne DB, Goldstein M (eds) Recent developments in Parkinson’s disease, vol 2. Florham Park, NJ. Macmillan Health Care Information, pp 153–163, 293–304

[CR24] Fanselow MS, Dong HW (2010). Are the dorsal and ventral hippocampus functionally distinct structures?. Neuron.

[CR25] Fenelon G, Alves G (2010). Epidemiology of psychosis in Parkinson’s disease. J Neurol Sci.

[CR26] Fenelon G, Mahieux F, Huon R, Ziegler M (2000). Hallucinations in Parkinson’s disease: prevalence, phenomenology and risk factors. Brain.

[CR27] Folstein MF, Folstein SE, McHugh PR (1975). “Mini-mental state”. A practical method for grading the cognitive state of patients for the clinician. J Psychiatr Res.

[CR28] Fox MD, Raichle ME (2007). Spontaneous fluctuations in brain activity observed with functional magnetic resonance imaging. Nat Rev Neurosci.

[CR29] Friedberg G, Zoldan J, Weizman A, Melamed E (1998). Parkinson Psychosis Rating Scale: a practical instrument for grading psychosis in Parkinson’s disease. Clin Neuropharmacol.

[CR30] Gallagher DA, Parkkinen L, O’Sullivan SS, Spratt A, Shah A, Davey CC, Bremner FD, Revesz T, Williams DR, Lees AJ, Schrag A (2011). Testing an aetiological model of visual hallucinations in Parkinson’s disease. Brain.

[CR31] Gama RL, Bruin VM, Tavora DG, Duran FL, Bittencourt L, Tufik S (2014). Structural brain abnormalities in patients with Parkinson’s disease with visual hallucinations: a comparative voxel-based analysis. Brain Cogn.

[CR32] Goetz CG (2009). Scales to evaluate psychosis in Parkinson’s disease. Parkinsonism Relat Disord.

[CR33] Goodale MA, Milner AD (1992). Separate visual pathways for perception and action. Trends Neurosci.

[CR34] Greicius MD, Krasnow B, Reiss AL, Menon V (2003). Functional connectivity in the resting brain: a network analysis of the default mode hypothesis. Proc Natl Acad Sci USA.

[CR35] Hacker CD, Perlmutter JS, Criswell SR, Ances BM, Snyder AZ (2012). Resting state functional connectivity of the striatum in Parkinson’s disease. Brain.

[CR36] Hamilton JM, Salmon DP, Galasko D, Raman R, Emond J, Hansen LA, Masliah E, Thal LJ (2008). Visuospatial deficits predict rate of cognitive decline in autopsy-verified dementia with Lewy bodies. Neuropsychology.

[CR37] Hamilton JM, Landy KM, Salmon DP, Hansen LA, Masliah E, Galasko D (2011). Early visuospatial deficits predict the occurrence of visual hallucinations in autopsy-confirmed dementia with Lewy bodies. Am J Geriatr Psychiatry.

[CR38] Hebart MN, Hesselmann G (2012). What visual information is processed in the human dorsal stream?. J Neurosci.

[CR39] Hoehn MM, Yahr MD (1967). Parkinsonism: onset, progression and mortality. Neurology.

[CR40] Ibarretxe-Bilbao N, Ramirez-Ruiz B, Tolosa E, Marti MJ, Valldeoriola F, Bargallo N, Junque C (2008). Hippocampal head atrophy predominance in Parkinson’s disease with hallucinations and with dementia. J Neurol.

[CR41] Ibarretxe-Bilbao N, Ramirez-Ruiz B, Junque C, Marti MJ, Valldeoriola F, Bargallo N, Juanes S, Tolosa E (2009). Differential progression of brain atrophy in Parkinson’s disease with and without visual hallucinations. J Neurol Neurosurg Psychiatry.

[CR42] Isaacson RL (2002). Unsolved mysteries: the hippocampus. Behav Cogn Neurosci Rev.

[CR43] Jenkinson M, Smith S (2001). A global optimisation method for robust affine registration of brain images. Med Image Anal.

[CR44] Jenkinson M, Bannister P, Brady M, Smith S (2002). Improved optimization for the robust and accurate linear registration and motion correction of brain images. Neuroimage.

[CR45] Kalaitzakis ME, Christian LM, Moran LB, Graeber MB, Pearce RK, Gentleman SM (2009). Dementia and visual hallucinations associated with limbic pathology in Parkinson’s disease. Parkinsonism Relat Disord.

[CR46] Krack P, Pollak P, Limousin P, Hoffmann D, Xie J, Benazzouz A, Benabid AL (1998). Subthalamic nucleus or internal pallidal stimulation in young onset Parkinson’s disease. Brain.

[CR47] Kwong KK, Belliveau JW, Chesler DA, Goldberg IE, Weisskoff RM, Poncelet BP, Kennedy DN, Hoppel BE, Cohen MS, Turner R (1992). Dynamic magnetic resonance imaging of human brain activity during primary sensory stimulation. Proc Natl Acad Sci USA.

[CR48] Ledberg A, Akerman S, Roland PE (1998). Estimation of the probabilities of 3D clusters in functional brain images. Neuroimage.

[CR49] Lim KO, Zipursky RB, Murphy GM, Pfefferbaum A (1990). In vivo quantification of the limbic system using MRI: effects of normal aging. Psychiatry Res.

[CR50] Liu AY, Rajji TK, Blumberger DM, Daskalakis ZJ, Mulsant BH (2013). Brain stimulation in the treatment of late-life severe mental illness other than unipolar nonpsychotic depression. Am J Geriatr Psychiatry.

[CR51] Matsui H, Udaka F, Tamura A, Oda M, Kubori T, Nishinaka K, Kameyama M (2006). Impaired visual acuity as a risk factor for visual hallucinations in Parkinson’s disease. J Geriatr Psychiatry Neurol.

[CR52] Merboldt KD, Hanicke W, Frahm J (1991). Diffusion imaging using stimulated echoes. Magn Reson Med.

[CR53] Merims D, Shabtai H, Korczyn AD, Peretz C, Weizman N, Giladi N (2004). Antiparkinsonian medication is not a risk factor for the development of hallucinations in Parkinson’s disease. J Neural Transm.

[CR54] Moskovitz C, Moses H, Klawans HL (1978). Levodopa-induced psychosis: a kindling phenomenon. Am J Psychiatry.

[CR55] Muller MJ, Greverus D, Dellani PR, Weibrich C, Wille PR, Scheurich A, Stoeter P, Fellgiebel A (2005). Functional implications of hippocampal volume and diffusivity in mild cognitive impairment. Neuroimage.

[CR56] Muller MJ, Greverus D, Weibrich C, Dellani PR, Scheurich A, Stoeter P, Fellgiebel A (2007). Diagnostic utility of hippocampal size and mean diffusivity in amnestic MCI. Neurobiol Aging.

[CR57] Nassi JJ, Callaway EM (2009). Parallel processing strategies of the primate visual system. Nat Rev Neurosci.

[CR58] Oertel V, Rotarska-Jagiela A, van de Ven VG, Haenschel C, Maurer K, Linden DE (2007). Visual hallucinations in schizophrenia investigated with functional magnetic resonance imaging. Psychiatry Res.

[CR59] Ogawa S, Tank DW, Menon R, Ellermann JM, Kim SG, Merkle H, Ugurbil K (1992). Intrinsic signal changes accompanying sensory stimulation: functional brain mapping with magnetic resonance imaging. Proc Natl Acad Sci USA.

[CR60] Olypher AV, Klement D, Fenton AA (2006). Cognitive disorganization in hippocampus: a physiological model of the disorganization in psychosis. J Neurosci.

[CR61] Onofrj M, Taylor JP, Monaco D, Franciotti R, Anzellotti F, Bonanni L, Onofrj V, Thomas A (2012). Visual Hallucinations in PD and Lewy body dementias: old and new hypotheses. Behav Neurol.

[CR62] Patenaude B, Smith SM, Kennedy DN, Jenkinson M (2011). A Bayesian model of shape and appearance for subcortical brain segmentation. Neuroimage.

[CR63] Pereira JB, Valls-Pedret C, Ros E, Palacios E, Falcon C, Bargallo N, Bartres-Faz D, Wahlund LO, Westman E, Junque C (2013). Regional vulnerability of hippocampal subfields to aging measured by structural and diffusion MRI. Hippocampus.

[CR64] Rabey JM (2009). Hallucinations and psychosis in Parkinson’s disease. Parkinsonism Relat Disord.

[CR65] Raichle ME, MacLeod AM, Snyder AZ, Powers WJ, Gusnard DA, Shulman GL (2001). A default mode of brain function. Proc Natl Acad Sci USA.

[CR66] Robbins TW, James M, Owen AM, Sahakian BJ, Lawrence AD, McInnes L, Rabbitt PM (1998). A study of performance on tests from the CANTAB battery sensitive to frontal lobe dysfunction in a large sample of normal volunteers: implications for theories of executive functioning and cognitive aging. Cambridge Neuropsychological Test Automated Battery. J Int Neuropsychol Soc.

[CR67] Robinson S, Basso G, Soldati N, Sailer U, Jovicich J, Bruzzone L, Kryspin-Exner I, Bauer H, Moser E (2009). A resting state network in the motor control circuit of the basal ganglia. BMC Neurosci.

[CR68] Rolls ET, Xiang JZ (2006). Spatial view cells in the primate hippocampus and memory recall. Rev Neurosci.

[CR69] Sanchez-Ramos JR, Ortoll R, Paulson GW (1996). Visual hallucinations associated with Parkinson disease. Arch Neurol.

[CR70] Satterthwaite TD, Wolf DH, Loughead J, Ruparel K, Elliott MA, Hakonarson H, Gur RC, Gur RE (2012). Impact of in-scanner head motion on multiple measures of functional connectivity: relevance for studies of neurodevelopment in youth. Neuroimage.

[CR71] Seeley WW, Menon V, Schatzberg AF, Keller J, Glover GH, Kenna H, Reiss AL, Greicius MD (2007). Dissociable intrinsic connectivity networks for salience processing and executive control. J Neurosci.

[CR72] Sridharan D, Levitin DJ, Menon V (2008). A critical role for the right fronto-insular cortex in switching between central-executive and default-mode networks. Proc Natl Acad Sci USA.

[CR73] Svanborg P, Asberg M (1994). A new self-rating scale for depression and anxiety states based on the Comprehensive Psychopathological Rating Scale. Acta Psychiatr Scand.

[CR74] Sykova E, Nicholson C (2008). Diffusion in brain extracellular space. Physiol Rev.

[CR75] Taylor DG, Bushell MC (1985). The spatial mapping of translational diffusion coefficients by the NMR imaging technique. Phys Med Biol.

[CR76] Van Dijk KR, Sabuncu MR, Buckner RL (2012). The influence of head motion on intrinsic functional connectivity MRI. Neuroimage.

[CR77] Vignal JP, Maillard L, McGonigal A, Chauvel P (2007). The dreamy state: hallucinations of autobiographic memory evoked by temporal lobe stimulations and seizures. Brain.

[CR78] Vincent JL, Patel GH, Fox MD, Snyder AZ, Baker JT, Van Essen DC, Zempel JM, Snyder LH, Corbetta M, Raichle ME (2007). Intrinsic functional architecture in the anaesthetized monkey brain. Nature.

[CR79] Vingerhoets FJ, Villemure JG, Temperli P, Pollo C, Pralong E, Ghika J (2002). Subthalamic DBS replaces levodopa in Parkinson’s disease: two-year follow-up. Neurology.

[CR80] Ward AM, Schultz AP, Huijbers W, Van Dijk KR, Hedden T, Sperling RA (2014) The parahippocampal gyrus links the default-mode cortical network with the medial temporal lobe memory system. Hum Brain Mapp. 35(3):1061–1073 10.1002/hbm.22234 PMC3773261 23404748

[CR81] Weissenbacher A, Kasess C, Gerstl F, Lanzenberger R, Moser E, Windischberger C (2009). Correlations and anticorrelations in resting-state functional connectivity MRI: a quantitative comparison of preprocessing strategies. Neuroimage.

[CR82] Yarnall AJ, Breen DP, Duncan GW, Khoo TK, Coleman SY, Firbank MJ (2014). Characterizing mild cognitive impairment in incident Parkinson disease: the ICICLE-PD study. Neurology.

[CR83] Yao N, Shek-Kwan Chang R, Cheung C, Pang S, Lau KK, Suckling J, Rowe J, Yu K, Ka-Fung Mak H, Chua SE, Ho SL, McAlonan GM (2014) The default mode network is disrupted in parkinson’s disease with visual hallucinations. Hum Brain Mapp (in press) 10.1002/hbm.22577 PMC4657500 24985056

[CR84] Zhang D, Raichle ME (2010). Disease and the brain’s dark energy. Nat Rev Neurol.

