# Supplementary material for: Multimodal MRI of the hippocampus in Parkinson’s disease with visual hallucinations
Source: Brain Struct Funct. 2014 Oct 7;221(1):287–300. doi: 10.1007/s00429-014-0907-5 (PMC4720723; doi:10.1007/s00429-014-0907-5)
Supplement: Supplementary file 1 — Supplementary material 1 (DOC 30 kb) [file 429_2014_907_MOESM1_ESM.doc]

**Supplementary table 1.** Post Hoc Tests

| Post hoc Test | HC vs PDnonVH | HC vs PDVH | PDnonVH vs PDVH | |
| --- | --- | --- | --- | --- |
| Hippocampal MD (R) | 0.965 | 0.004^**^ | | 0.008^**^ |
| Hippocampal MD (L) | 0.793 | 0.019^*^ | | 0.075 |
| PAL Total trials adjusted | 0.038^*^ | 0.047^*^ | | 0.992 |
| PAL First trial memory score | 0.064 | 0.006^**^ | | 0.452 |
| PAL Total errors adjusted | 0.073 | 0.066 | | 0.967 |

Post hoc multiple comparisons were calculated using Tukey's HSD (honestly significant difference) test.

^*^p <0.05.

^**^p <0.01.
