# Supplementary material for: Multimodal MRI of the hippocampus in Parkinson’s disease with visual hallucinations
Source: Brain Struct Funct. 2014 Oct 7;221(1):287–300. doi: 10.1007/s00429-014-0907-5 (PMC4720723; doi:10.1007/s00429-014-0907-5)
Supplement: Supplementary file 2 — Supplementary material 2 (DOC 27 kb) [file 429_2014_907_MOESM2_ESM.doc]

**Supplement table 2** Pearson correlation coefficients between FC measures and cognitive scores in PDVH group.

| PAL score | Total trials adjusted | First trial memory score | Total errors adjusted | Stages completed |
| --- | --- | --- | --- | --- |
| Right occipital gyrus | -0.719* | 0.546 | -0.630 | 0.584 |
| Right medial temporal lobe | -0.772* | 0.592 | -0.620 | 0.709* |

Correlation coefficients were controlled for age and visual accuracy scores.

*p<0.05.
