# Supplementary figures and images for: Multimodal MRI of the hippocampus in Parkinson’s disease with visual hallucinations
Source: Brain Struct Funct. 2014 Oct 7;221(1):287–300. doi: 10.1007/s00429-014-0907-5 (PMC4720723; doi:10.1007/s00429-014-0907-5)

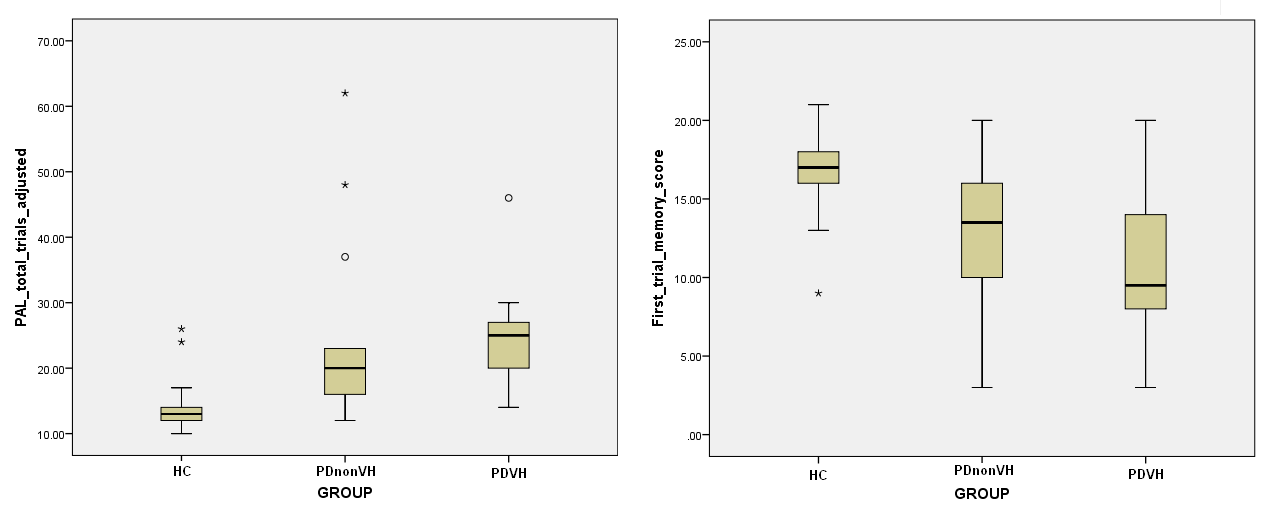

Supplement: Supplementary file 3 — PAL scores of HC, PDnonVH and PDVH groups. Supplementary material 3 (TIFF 1,966 kb) [file 429_2014_907_MOESM3_ESM.tif]

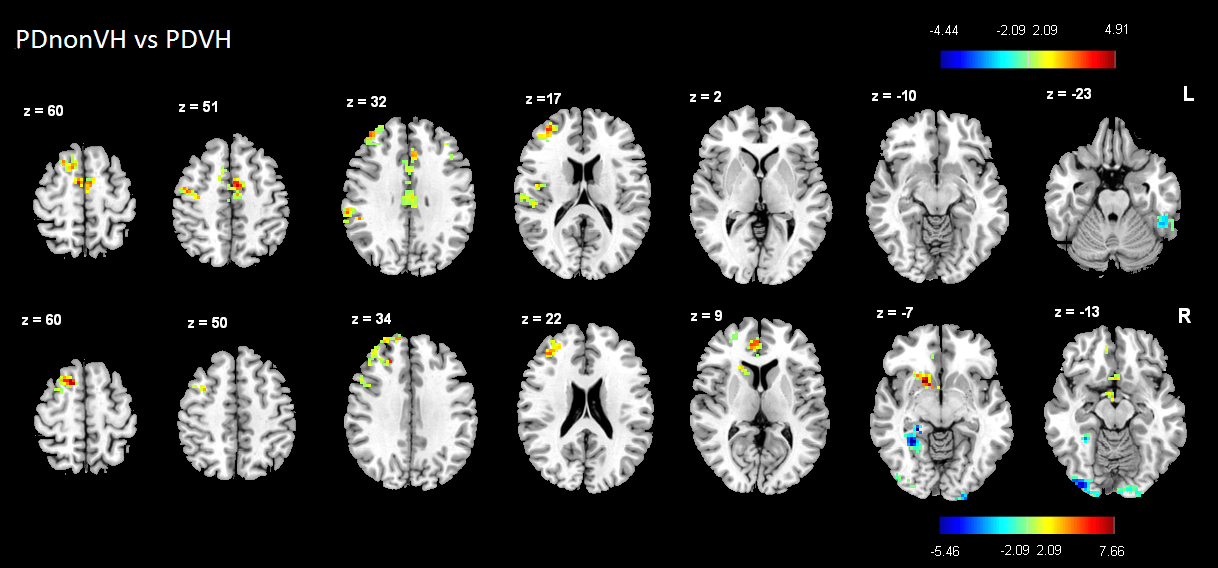

Supplement: Supplementary file 4 — Group differences between PDnonVH and PDVH in hippocampal functional connectivity. Statistical results are superimposed on the standard Montreal Neurological Institute template (x coordinates are reported) (mm) at the given threshold corrected for multiple comparisons (Monte-Carlo Simulation, cluster size=1701 mm3 (63 voxels), in comparison between PDnonVH and PDVH, T>2.09 (or T<−2.09)). Age, MMSE scores, levodopa-equivalent dosage, head motion and working memory scores were entered as covariables. In each panel, blue indicates relatively lower functional connectivity in PDVH compared to PDnonVH; red indicates the reverse. The right side of the figure is the left side of the brain. L = functional connectivity with left hippocampus; R = functional connectivity with the right hippocampus. Supplementary material 4 (TIFF 2,027 kb) [file 429_2014_907_MOESM4_ESM.tif]
